# Reduced Dynamic Interactions Within Intrinsic Functional Brain Networks in Early Blind Patients

**DOI:** 10.3389/fnins.2019.00268

**Published:** 2019-03-22

**Authors:** Xianglin Li, Ailing Wang, Junhai Xu, Zhenbo Sun, Jikai Xia, Peiyuan Wang, Bin Wang, Ming Zhang, Jie Tian

**Affiliations:** ^1^Department of Medical Imaging, The First Affiliated Hospital of Xi’an Jiaotong University, Xi’an, China; ^2^Medical Imaging Research Institute, Binzhou Medical University, Yantai, China; ^3^Department of Clinical Laboratory, Yantai Affiliated Hospital of Binzhou Medical University, Yantai, China; ^4^Tianjin Key Laboratory of Cognitive Computing and Application, School of Artificial Intelligence, College of Intelligence and Computing, Tianjin University, Tianjin, China; ^5^Department of Radiology, Yantai Affiliated Hospital of Binzhou Medical University, Yantai, China; ^6^School of Life Sciences and Technology, Xidian University, Xi’an, China

**Keywords:** early blindness, effective connectivity, spectral dynamic causal modeling, intrinsic brain networks, brain plasticity

## Abstract

Neuroimaging studies in early blind (EB) patients have shown altered connections or brain networks. However, it remains unclear how the causal relationships are disrupted within intrinsic brain networks. In our study, we used spectral dynamic causal modeling (DCM) to estimate the causal interactions using resting-state data in a group of 20 EB patients and 20 healthy controls (HC). Coupling parameters in specific regions were estimated, including the medial prefrontal cortex (mPFC), posterior cingulate cortex (PCC), and inferior parietal lobule (IPC) in the default mode network (DMN); dorsal anterior cingulate cortex (dACC) and bilateral anterior insulae (AI) in the salience network (SN), and bilateral frontal eye fields (FEF) and superior parietal lobes (SPL) within the dorsal attention network (DAN). Statistical analyses found that all endogenous connections and the connections from the mPFC to bilateral IPCs in EB patients were significantly reduced within the DMN, and the effective connectivity from the PCC and lIPC to the mPFC, and from the mPFC to the PCC were enhanced. For the SN, all significant connections in EB patients were significantly decreased, except the intrinsic right AI connections. Within the DAN, more significant effective connections were observed to be reduced between the EB and HC groups, while only the connections from the right SPL to the left SPL and the intrinsic connection in the left SPL were significantly enhanced. Furthermore, discovery of more decreased effective connections in the EB subjects suggested that the disrupted causal interactions between specific regions are responsive to the compensatory brain plasticity in early deprivation.

## Introduction

The recognition of objects could be manipulated through the coordinated cross-modal interactions of different modalities, such as vision, touch and audition (Amedi et al., 2005; Dormal et al., 2018). Deprivation of one sensory modality could give us a chance to explore the plasticity changes of the cognitive functions (Jiang et al., 2015). Two hypotheses have been raised to explain the plasticity in early blindness. The first hypothesis indicated a kind of maladjustment caused by the early blindness. When the sensory information is manipulated, there is a decrease in processing capacities by the early visual deprivation (Jiang et al., 2015). The other hypothesis is based on the compensatory explanation, in which the blind patients exhibit a superior ability in retained sensory modalities (Pascual-Leone et al., 2005). The early visual deprivation has been demonstrated to cause the structural and functional remodeling or reorganization in both intact and deprived sensory cortices (Bauer et al., 2017, 2018; Hou et al., 2017). Furthermore, impaired cognitive performances have been discovery in specific brain regions or networks (Dormal et al., 2018; Manescu et al., 2018; Vercillo et al., 2018). The baseline metabolism and blood flow in specific cortices have been suggested to show a significant increase in early blind (EB) patients (Viski et al., 2016). Functional correlations were observed between the subregions of the visual cortex in the retinotopic pattern in EB subjects at rest (Bock et al., 2015). Furthermore, EB patients also exhibit disrupted functional connections and stronger parietal and auditory networks compared with sighted subjects (Boldt et al., 2014; Hou et al., 2017; Abboud and Cohen, 2019). Therefore, EB patients showed a compensatory pattern in the primary sensory networks, while the other brain networks related to cognition might be disrupted due to the early deprivation.

The resting-state technique on functional magnetic resonance imaging (fMRI) has been proved to be powerful in revealing the abnormalities of intrinsic functional connections. The default mode network (DMN) is the most prominent system in the resting state (Mccormick and Telzer, 2018; Prestel et al., 2018), which consists of the medial prefrontal cortex (mPFC), posterior cingulate cortex (PCC), and bilateral inferior parietal lobule (IPL), and some brain areas in the temporal lobe (Sharaev et al., 2016). By applying advanced brain network analyses to the resting-state data, abnormalities of the functional integration in EB patients have been examined in many studies, reflecting the statistical dependencies between distant brain regions (Heine et al., 2015; Sabbah et al., 2016). However, correlation parameters can only calculate the statistical dependency between two regions, while the causal interactions cannot be estimated. Moreover, DMN regions have been observed to be hyperactivated in many cognitive tasks, like autobiographical information retrieval, mind-wandering, emotional processing, and spontaneous cognition (Turkheimer et al., 2015; Hu et al., 2017).

In addition to the DMN, two other distinct brain networks (ventral and dorsal) play a vital role in EB subjects in the allocation of attention (Jimenez et al., 2016). The ventral network consists of the temporoparietal junction, anterior cingulate cortex (ACC), and anterior insulae (AI), which has been suggested to be responsible for stimulus-driven attention (Jimenez et al., 2016; Liu et al., 2017; Wang et al., 2018). The other dorsal network, constituted of the lateral frontal eye field (FEF) as well as the bilateral superior parietal lobes (SPL), is correlated with the voluntary, sustained orienting of attention (Corbetta and Shulman, 2011; Tsvetanov et al., 2016). Recent studies have highlighted the dysfunction in the ventral network in subjects with disorders, but not in the dorsal network (White et al., 2013; Wynn et al., 2015). The ventral attention network can also be called the “salience network” (SN). Pathophysiological studies have demonstrated that EB subjects with dysfunctions could result in the incorrect assigning of salience (Palaniyappan and Liddle, 2012). Therefore, the application of the resting-state fMRI approach can be potentially powerful in the assessment of attentional deficits in EB patients when we intend to examine the relative contributions of these two networks.

Dynamic causal modeling (DCM) is an appropriate approach to examine the causal influence, which is designed to calculate the effective connectivity changes underlying human responses based on Bayesian analysis. When one specific model is constructed (including specific brain regions, directed connections, and modulations), the parameters are estimated based on the observed data and model structures. Dynamic causal modeling is model-based, and shows great advantages compared to the functional connectivity or data-driven effective connectivity like the Granger causality, as DCM allows different hypotheses to be tested, which could capture the functional brain architectures corresponding to a specific hypothesis (Friston et al., 2017). Many studies have been reported by applying DCM to fMRI data and Magnetoencephalography/electroencephalography (MEG/EEG) (Chahine et al., 2017; Yang et al., 2017; He and Johnson, 2018; Van De Steen et al., 2019). Recently, a new version of DCM-spectral DCM was developed to estimate the intrinsic effective connections from resting-state data using the cross spectra of the signals through a deterministic model (Friston et al., 2014). The cross-spectra can be considered as a more complete measure of functional connections (Park et al., 2018). Spectral DCM renders the model essentially in a deterministic way to get rid of the heavy load in estimating the random fluctuations in neural states. Therefore, spectral DCM makes DCM in resting-state fMRI slightly simpler and does not require a bilinear term accounting for condition-specific effects (Razi et al., 2015). Furthermore, spectral DCM is intended to simply compare the endogenous coupling between different groups (healthy controls vs. patients), so it only estimates the time-invariant parameters of the cross spectra. As the frequency domain is used in estimating the effective connectivity, the high computational efficiency and stable estimation make spectral DCM significantly powerful to compare the couplings and directionality in the intrinsic brain networks between groups of subjects, like patients and healthy cohorts (Li et al., 2017). Therefore, this is the reason for choosing the spectral DCM approach in this study.

Previous studies have suggested that the EB subjects showed a disruption of functional connectivity both in the visual cortex and other brain regions located in other cognitive brain networks (Liu et al., 2017; Dormal et al., 2018; Abboud and Cohen, 2019). Early deprivation could trigger the functional preference for the selective auditory recruitment in the cognitive brain networks (Dormal et al., 2018) and a reduction of the interhemispheric functional connectivity in the cognitive regions (Hou et al., 2017). Although the disruption of the functional interactions among the regions from the visual cortex and the cognitive brain networks, like the DMN and SN, it remains unclear how the directed interactions between these regions changed in the EB subjects. The purpose of our study is to examine the changes in the effective connectivity within three high-order cognitive networks between EB patients and healthy controls (HC) by estimating the corresponding parameters with a newly developed spectral DCM. In this study, we had one hypothesis: early deprivation could affect the dynamic interactions between regions within three brain networks. Furthermore, the strength of the effective connectivity between regions in all three networks might be decreased due to the early deprivation. The spectral DCM was used to explore the causal neuronal influences within the intrinsic functional networks (DMN, SN and DAN) in a sample of 20 EB patients and 20 matched HC. Firstly, an independent component analysis was made to the resting-state functional images to generate the DMN, SN and DAN components. Then, the spectral DCM was employed to compute the effective connectivity within DMN, SN, and DAN of EB patients and HC. A Bayesian model selection procedure was adopted to determine the optimal DCM model at the group level. Statistical analyses were further conducted to compare the differences in the effective connectivity parameters within DMN, SN, and DAN between EB and HC subjects.

## Materials and Methods

### Participants

Twenty EB subjects and 20 sighted subjects participated in this study. The mean age was 22.3 ± 1.4 years for the blind group, and 20.7 ± 1.2 years for the sighted group; there was no significant difference between both groups. The blindness of these subjects was evaluated and diagnosed by two professional ophthalmologists from Yantai Affiliated Hospital of Binzhou Medical University based on the retinal pathology. All the EB subjects had the same categories of blindness, mainly caused by cataracts and retinal pigment degeneration (Cataract: 8; Retinal pigment degeneration: 12). Table 1 shows the detailed information for all EB subjects. All subjects were right-handed, and no subject had neurological problems except for the visual deprivation. After hearing a detailed explanation on the study, all subjects gave written informed consent. This study was carried out in accordance with the recommendations of Institutional Review Board of Binzhou Medical University. The protocol was approved by the Institutional Review Board of Binzhou Medical University. All subjects gave written informed consent in accordance with the Declaration of Helsinki after hearing detailed explanation about the study.

**Table 1:** Demographic information for the early blind (EB) groups.

Onset of visual	Number of		Education
deprivation	subjects	Age (Year)	level (Year)
At birth	15	22.14 ± 1.43	13.27 ± 1.16
4 years old	1	24.3	11
5 years old	3	22.27 ± 0.83	13.67 ± 0.58
6 years old	1	22.9	12

*Onset of visual deprivation means the age of the EB subject sight loss*.

### Data Acquisition

The functional images were scanned using a 3.0 T Siemens Skyra scanner with a 32-channel head coil at Yantai Affiliated Hospital of Binzhou Medical University. Participants were instructed to relax and keep their eyes closed without thinking about anything in particular. A high-resolution structural image was collected using a T1 weighted 3D MPRAGE sequence (repetition time (TR) = 1900 ms, echo time (TE) = 2.52 ms, inversion time (TI) = 1100 ms, voxel size = 1 × 1 × 1 mm^3^, matrix size = 256 × 256, flip angle (FA) = 90°). A gradient-echo planar imaging (EPI) sequence (TR = 2000 ms, TE = 30 ms, voxel size = 3.1 × 3.1 × 4.0 mm^3^, matrix size = 64 × 64, slices = 33, slices thickness = 4 mm, slices gap = 0.6 mm, FA = 9°) was used for functional data collection. Earplugs and foam pads were used to reduce the scanner noise and head motion.

### Data Preprocessing

SPM12 was used for the data preprocessing^1^. For each participant, the first 10 functional images were first removed to allow for participants’ adaptation to the environment and equilibration effects. The remaining data were processed by a slice timing correction and head motion correction by a realign analysis. No participant was excluded, as all participants’ head movements were not larger than 1.5 mm and 1 degree. The high-resolution structural image was coregistered with the functional images and subsequently segmented into the gray matter, white matter and cerebrospinal fluid (CSF). The generated spatial parameters from the segmentation procedure were then applied to spatially normalize the realigned images to 3 × 3 × 3 mm^3^ in the Montreal Neurological Institute (MNI) space. Finally, the functional images were smoothed with a full-width at half-maximum Gaussian filter (FWHM = 4 mm) to attenuate spatial noise. Several variances were regressed out with the temporal derivatives with the linear regression, including six head motion parameters and averaged signals from the CSF and white matter. Specifically, the global signal regression was not performed in this study, as global signals are thought to be irrelevant to non-neural noise and should not be regressed out (Chen et al., 2012).

### Group Independent Component Analysis (Group ICA)

To extract the regions for the subsequent DCM analysis, the DMN, SN, and DAN components were first identified for each subject. Group ICA was performed to decompose the resting state functional images into spatially independent components (ICs) using the GIFT software^2^ after data preprocessing. First, the number of the optimal components were estimated using the minimum description length criteria (MDL) (Li et al., 2007). The number of components in our experiment were 41. Therefore 41 independent spatial components were produced using the infomax algorithm. For each participant, the corresponding spatial maps were generated through a back-reconstruction step.

The DMN, SN, and DAN templates were generated by the WFU PickAtlas Tool 3.0 to identify the DMN, SN, and DAN components (Calhoun et al., 2008). In our study, we generated the DMN template by including the posterior cingulate cortex, bilateral precuneus, superior medial frontal cortex, and inferior parietal lobe. A multiple spatial regression analysis between the template and ICs was then conducted to sort all the spatial components. By comparing the regression coefficients, the component that fit best with the template with the greatest one was identified as the DMN component. The one-sample *t*-test was used to generate a group-mean pattern (*p* < 0.001, uncorrected, cluster size = 10 voxels). For the SN and DAN components, the same procedure was performed by creating the SN and DAN templates. To keep the consistency of the locations of each ROI between two groups, the Euclidean distance was also calculated between the individual ROI and group-mean ROI.

### Definition of Region of Interest (ROI)

For each subject, all the specific ROIs were selected based on the spatial map of the individual component representing three brain networks. Based on the regions identified above, four ROIs were defined for DMN: the mPFC, PCC, and bilateral parietal cortex (lIPC and rIPC). We defined three regions: dorsal cingulate cortex (dACC), left anterior (lAI), and right anterior insulae (rAI) for the salience network. Additionally, the DAN included the bilateral frontal eye fields (lFEF, rFEF) and bilateral superior parietal lobes (lSPL, rSPL). The detailed information of ROIs is shown in Table 2.

**Table 2:** Coordinates of the regions of interest used in spectral dynamic causal modeling (DCM) analysis.

Region	Brain network	MNI coordinates		
		*x*	*y*	*z*
mPFC	DMN	3	54	-2
lIPC	DMN	-49	-62	32
rIPC	DMN	47	-68	35
PCC	DMN	0	-52	26
dACC	SN	0	-10	40
lAI	SN	-43	-11	-1
rAI	SN	43	-11	-1
lFEF	DAN	-24	-15	66
rFEF	DAN	28	-10	58
lSPL	DAN	-24	-55	72
rSPL	DAN	24	-55	72

*mPFC, medial prefrontal cortex; IPC, inferior parietal lobule; PCC, posterior cingulate cortex; dACC, dorsal cingulate cortex; AI, anterior insulae; FEF, frontal eye field; SPL, superior parietal lobe; DMN, default mode network; SN, salience network; DAN, dorsal attention network; l, left; r, right*.

All the subject-specific ROIs were defined as spheres with a radius of 8 mm centered at the local maximum from the identified DMN, DAN, and SN components for each subject. We also included a white matter mask to remove the influence of the white matter. This procedure would ensure the further DCM analysis was conducted on the consistent regions identified as one functionally connected network for each subject. Finally, time series were extracted from all ROIs as the residuals of the general linear model, which was constructed by the following regressors: the head motion parameters, cosine basis functions to model the aliased respiratory and cardiac signals, one constant regressor to model the baseline and a high-pass filter of 1/128 Hz to regress the ultraslow fluctuations.

### Spectral Dynamic Causal Modeling

To estimate the effective connectivity parameters in specific regions, the spectral DCM analysis was conducted for the defined nodes of all three networks using DCM12 in SPM12. The spectral DCM is designed to estimate the intrinsic effective connectivity from resting state fMRI images with the cross-spectra of the signals. The cross spectral in the frequency domain could be considered as a more complete measure of functional connectivity (Park et al., 2018). In theory, the spectral DCM is distinct from stochastic DCM, which can estimate the coupling parameters among coupled populations of neurons in the frequency domain. It calculates the spectral measure by using a neuronally plausible power-law model among measured responses, and the time-invariant parameters. It models the observed functional connectivity between nodes with their second-order statistics instead of the neural signals under a deterministic assumption. In other words, spectral DCM could estimate the covariance of the random fluctuations from previous experiences to create the complex cross spectra among measured responses (Razi et al., 2015).

The expected cross spectra can be generated by the following model:

$$g_{y}\mathrm{(\omega ,}\;\mathrm{\theta )}\;=\;{\left|K\mathrm{(\omega )}\right|}^{2}\;g_{v}\mathrm{(\omega ,}\;\;\mathrm{\theta )}\;+\;g_{e}\mathrm{(\omega ,}\;\;\mathrm{\theta )}$$

Here, g_y_(ω,θ) represents the predicted cross spectra that can be estimated. K(ω) is a function of the effective connection, which is the Fourier transform of the system’s Volterra kernel.

For each participant, a fully connected model was constructed with reciprocal connectivity between any pair of all ROIs by a Bayesian network discovery analysis (Friston et al., 2011). For resting-state fMRI data, no exogenous input was added to the model construction, then we made a parameter estimation for all models. Unlike the stochastic DCM, the convolution kernel representation in the spectral DCM was transformed to a spectral representation in the frequency domain. An estimation procedure was applied to characterize the spectral densities over frequencies for all estimated parameters within the DMN, SN, and DAN. Bayesian model selection (BMS) was finally performed with a *post hoc* optimization method to estimate the optimal model with the best balance between complexity and accuracy. The BMS was conducted on both groups separately, as we assumed both groups did not share the same model structure. The corresponding parameters for the best model were also estimated.

### Statistical Analysis

After the parameter estimation was completed for the optimal model, one-sample *t*-tests were conducted to examine whether the effective connectivity between regions was significant for both EB and HC groups. Multiple comparison corrections were made on the results of one-sample *t*-tests at *p* < 0.05 by applying the false discovery rate (FDR) procedure. To examine the abnormalities of the effective connectivity within all three networks between EB and HC cohorts, the coupling parameters from both groups were tested using the two-sample *t*-tests. A correction for FDR was used to determine the results of two-sample *t*-test at *p* < 0.05.

## Results

### Independent Component Analysis

**Figure 1:**
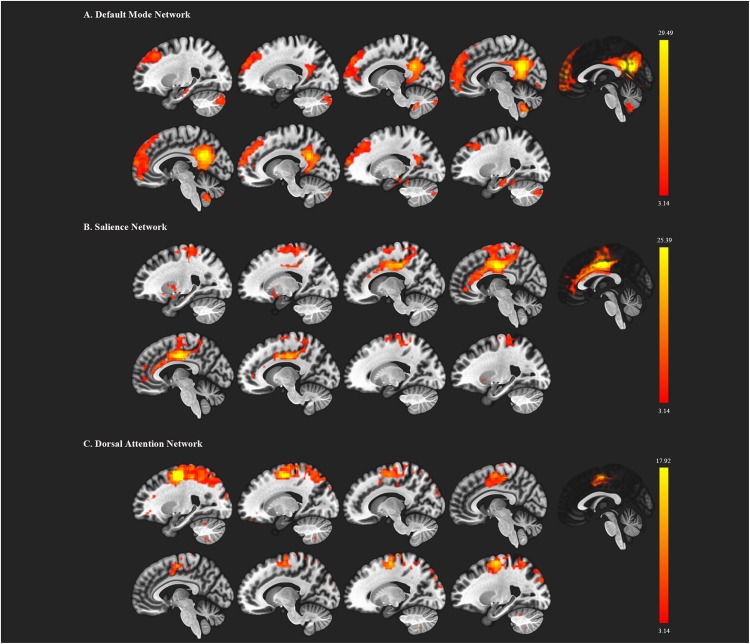
Functional brain networks chosen from the independent component analysis. **(A)** represents the default mode network (DMN); **(B)** represents the salience network; **(C)** represents the dorsal attention network. The red color regions correspond to the threshold *z*-value.

We first decomposed the separated spatial patterns by conducting the group ICA on the resting-state functional images. Forty-one independent components were generated according to the MDL criterion, and the generated components were sorted by their correlations with the DMN, SN, and DAN templates. The independent components that showed a best fit with all three templates were identified as the DMN, SN, and DAN components. Subject-specific nodes were defined as 8-mm spheres centered at the peak values of all ROIs for each subject. Figure 1 shows the profiles of the DMN, SN, and DAN components. Based on the group ICA analysis, four ROIs were defined for DMN, including the mPFC, PCC, bilateral IPC; three ROIs for SN: dACC, lAI, and rAI, and four bilateral ROIs for DAN: lEFF, rFEF, lSPL, and rSPL. The coordinates of all selected ROIs are shown in Table 2. The statistical analysis on the Euclidean distance suggested there was no significant difference in the Euclidean distance between two groups, indicating the consistency of the locations of the subject-specific ROI.

### DCM Analysis and Effective Connectivity of DMN, SN, and DAN

**Figure 2:**
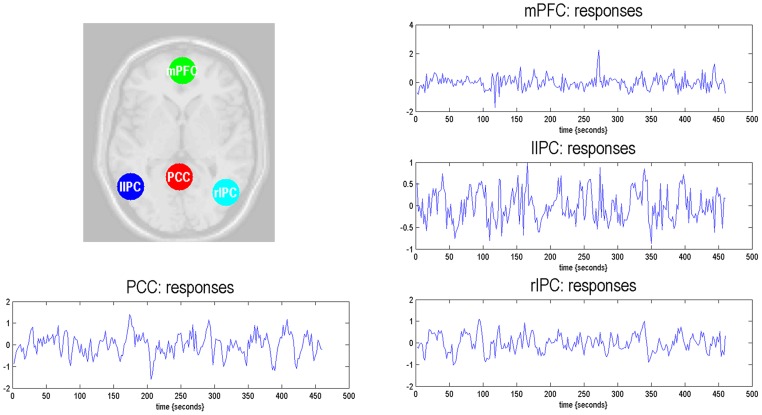
Locations of the representative regions within the DMN. The DMN regions are identified from the independent component analysis. The principal eigenvariates are extracted from the corresponding time series of regions.

**Figure 3:**
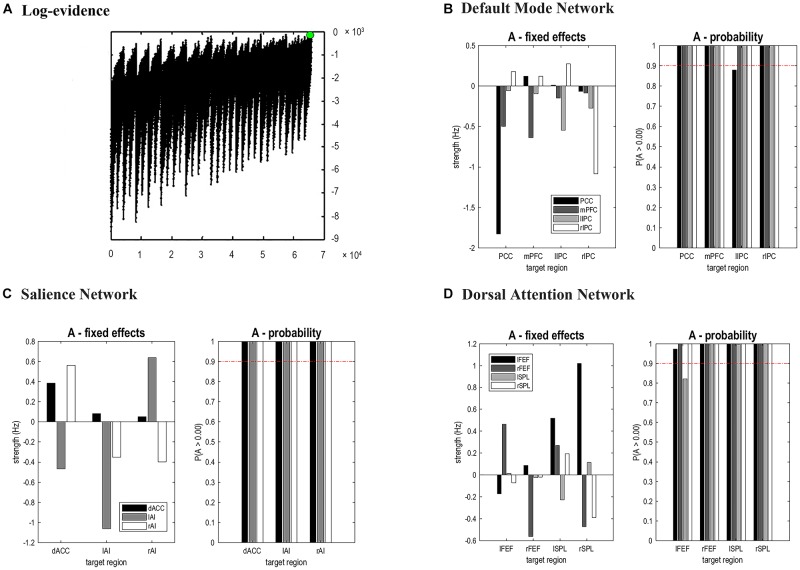
Results of Bayesian Model Selection analysis and estimations of the fixed effects. For DMN, the fully connected model showed the highest evidence **(A)**, which suggests that the full model was the best explanation for our data. **(B–D)** showed the estimated fixed effects and posterior probabilities of these effective connectivity parameters for DMN, SN, and DAN. The red dashed line depicted the 95% threshold. mPFC, medial prefrontal cortex; IPC, inferior parietal lobule; PCC, posterior cingulate cortex; dACC, dorsal cingulate cortex; AI, anterior insulae; FEF, frontal eye field; SPL, superior parietal lobe; l, left; r, right.

The time series of all ROIs were first extracted for each subject. For illustration, we extracted the principal eigenvariates from the corresponding time series of the selected regions within the DMN, as shown in Figure 2. The generalized fitting procedure suggested that the observed spontaneous fMRI signals can be modeled by the conditional expectations in the neuronal activations. For each participant, a Bayesian model reduction procedure was used to search for the optimal model from the model space. The detailed results of the estimation are shown in Figure 3. Finally, 6536 models were constructed with all possible combinations using four ROIs. The log-evidence of all models for DMN is shown in Figure 3A, suggesting a model with more connections shared larger evidence. In this study, we found that the fully connected model was optimal with the most significant efficiency. The meant that our resting-state fMRI data could be stimulated by the fully connected model. There was a deduction of the log-evidence when any connection was removed.

**Figure 4:**
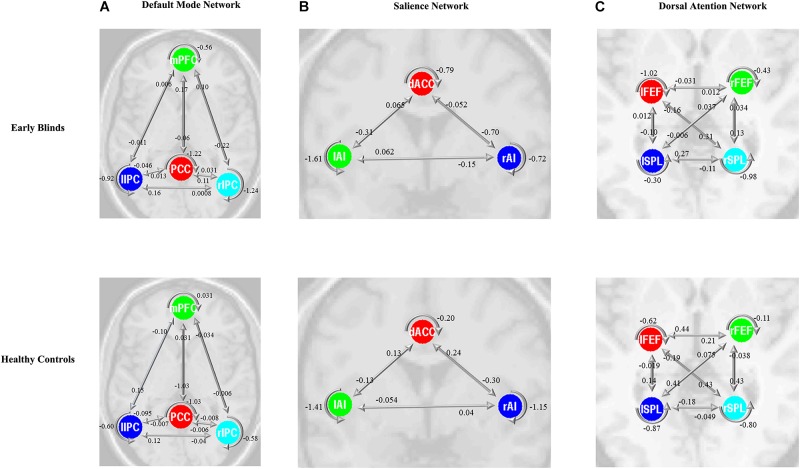
Bayes model average parameters of intrinsic brain networks (**A**, default mode network; **B**, salience network; and **C**, dorsal attention network) for early blinds and healthy controls (HCs). The number behind the colored lines represents the parameters of the effective connectivity between ROIs. mPFC, medial prefrontal cortex; IPC, inferior parietal lobule; PCC, posterior cingulate cortex; dACC, dorsal cingulate cortex; AI, anterior insulae; FEF, frontal eye field; SPL, superior parietal lobe; l, left; r, right. Double arrow means reciprocal connections.

The same DCM analyses were conducted for SN and DAN. As expected for DMN, the best models were the fully connected models for SN and DAN. In the EB and HC groups, the coupling parameters of the optimal model for each network were calculated by the Bayes model average analysis. Figure 3B–D shows the estimated fixed effects and the posterior probabilities for DMN, SN, and DAN in one typical subject. The Bayes model average parameters were then estimated for all subjects, as shown in Figure 4. Most of the coupling parameters within the DMN for EB and HC groups were observed to be significant (*p* < 0.05, df = 19), except the connections from PCC and mPFC to the right IPC and from bilateral IPC to PCC in the HC group; the connection from the left IPC to the other three regions and from the mPFC to the left IPC in the EB subjects, as shown in Table 3. All the effective connections within the SN were found to be significant in both the EB and NC groups (*p* < 0.05, df = 19). For the DAN, no significance was found except in one effective connection (left SPL→ left FEF) in the HC group, and in two connections (left SPL→ left FEF and left FEF→ right FEF). Tables 4, 5 showed the details of the average parameters of SN and DAN.

**Table 3:** Bayes model average parameters of the DMN for healthy controls (HCs) and EBs.

Groups		From PCC	From mPFC	From rIPC	From lIPC
HCs	To PCC	-1.03**	-1.03**	-0.0085	-0.0074
	To mPFC	0.031*	0.031*	-0.034*	-0.10**
	To rIPC	-0.0059	-0.0059	-0.58**	-0.040*
	To lIPC	-0.095*	0.15**	0.12*	-0.60**
EBs	To PCC	-1.22**	-0.061*	0.031*	0.013
	To mPFC	0.17**	-0.56**	0.10*	0.0064
	To rIPC	0.11*	-0.22**	-1.24**	0.00078
	To lIPC	-0.046*	-0.011	0.16**	-0.92**

*The averaged parameters represented the effective connectivity between regions within the DMN. The positive value between from node A to node B showed a positive modulation, suggesting that node A caused an increase in the rate of change of node B’s signal and vice versa. mPFC, medial prefrontal cortex; IPC, inferior parietal lobule; PCC, posterior cingulate cortex; DMN, default mode network; l, left; r, right. ^∗^means *p* < 0.05; ^∗∗^means *p* < 0.001*.

**Table 4:** Bayes model average of the salience network for HCs and EBs.

Groups		From dACC	From lAI	From rAI
HCs	To dACC	-0.20**	0.13**	0.24**
	To lAI	-0.13**	-1.41**	-0.054*
	To rAI	-0.30**	0.04*	-1.15**
				
EBs	To dACC	-0.79**	0.065*	-0.052*
	To lAI	-0.31**	-1.61**	0.062*
	To rAI	-0.70**	-0.15**	-0.72**

*The averaged parameters represented the effective connectivity between regions within the salience network. The positive value between from node A to node B showed a positive modulation, suggesting that node A caused an increase in the rate of change of node B’s signal and vice versa. dACC, dorsal cingulate cortex; AI, anterior insulae; SN, salience network; l, left; r, right. ^∗^means *p* < 0.05; ^∗∗^means *p* < 0.001*.

**Table 5:** Bayes model average parameters of the dorsal attention network for HCs and EBs.

Groups		From lFEF	From rFEF	From lSPL	From rSPL
HCs	To lFEF	-0.62**	0.44**	-0.019	-0.19**
	To rFEF	0.21**	-0.11*	0.075*	-0.038*
	To lSPL	0.14**	0.41**	-0.87**	-0.18**
	To rSPL	0.43**	0.43**	-0.049*	-0.80**
					
EBs	To lFEF	-1.02**	-0.031*	0.012	-0.16**
	To rFEF	0.012	-0.43**	0.037*	0.034*
	To lSPL	-0.10*	-0.0062	-0.30**	0.27**
	To rSPL	0.31**	0.13**	-0.11*	-0.98**

*The averaged parameters represented the effective connectivity between regions within the dorsal attention network. The positive value between from node A to node B showed a positive modulation, suggesting that node A caused an increase in the rate of change of node B’s signal and vice versa. FEF, frontal eye field; SPL, superior parietal lobe; DAN, dorsal attention network; l, left; r, right. ^∗^means *p* < 0.05; ^∗∗^means *p* < 0.001*.

### Alteration of the Effective Connectivity Within DMN, SN, and DAN

**Figure 5:**
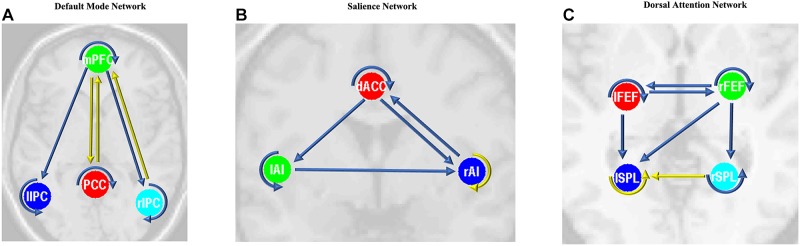
Significant effective connections within the default mode network **(A)**, salience network **(B)**, and dorsal attention network **(C)** between the early blind (EB) and healthy control (HC) subjects. The yellow line indicates the coupling parameter of the effective connectivity is enhanced by comparing the EB group to the HC group, while the blue line indicates reduced strength. The arrow represents the direction of the effective connectivity. mPFC, medial prefrontal cortex; IPC, inferior parietal lobule; PCC, posterior cingulate cortex; dACC, dorsal cingulate cortex; AI, anterior insulae; FEF, frontal eye field; SPL, superior parietal lobe; l, left; r, right.

After the parameters within the DMN, SN, and DAN were estimated, the two-sample *t*-tests analysis between the EB and HC groups showed that EB subjects showed significantly enhanced effective connectivity from PCC and lIPC to mPFC, and from mPFC to PCC (*p* < 0.05, df = 18). Besides all endogenous connections, the effective connectivity from the mPFC to bilateral IPC were significantly decreased (Figure 5A). For the SN, almost all the significant effective connections between the EB and NC groups were reduced, except the intrinsic connection in the right AI including the connections from the dACC to bilateral AI, right AI to dACC, and effective connections from the left AI to right AI (*p* < 0.05, df = 18; Figure 5B). As shown in Figure 5C, more significant effective connections were found to be reduced within the DAN between EB and HC groups, which included the connections from the left FEF to right FEF, and left SPL, the connections from the right FEF to all the other three nodes, and the intrinsic connections in the rSPL (*p* < 0.05, df = 18) Only the connections from the right SPL and itself to the left SPL were observed to be enhanced. Figure 5 shows the significantly enhanced and reduced effective connections within the DMN, SN, and DAN between EB and HC subjects.

## Discussion

In this study, the spectral DCM was used to compute the effective connections within the DMN, SN and DAN with resting-state fMRI data. The intrinsic brain networks were identified using the group ICA, and several nodes were defined for further DCM analyses. The fully connected model was found to be the optimal explanation for all three networks for our functional data. The one-sample *t*-test analysis suggested that most effective connections within all three networks were significant in both the EB and HC groups. Two-sample *t*-test analyses found that reduced coupling parameters of the effective connectivity for each network were observed by comparing the EB patients to the HC subjects, suggesting that there was a disruption of the effective integration within the resting-state brain networks in EB subjects.

### Effective Connectivity Analysis Within DMN

Increasing attention has been paid to the functional interactions within the DMN in normal and disordered people (Fang et al., 2016; Van Den Heuvel and Thomason, 2016; Khalili-Mahani et al., 2017). Directed functional interactions within the DMN regions have been explored using a series of approaches, such as the partial coherence analysis (Silfverhuth et al., 2011), Granger causality analysis (GCA) (Zhou et al., 2011) and Bayesian networks (Wu et al., 2011). One latest study found a reduction of interhemispheric functional connectivity in early blindness using the resting-state fMRI (Hou et al., 2017), suggesting the disruption of the functional connections by early deprivation. However, the DCM method was distinct from the above approaches in theory, because the DCM was model-based by constructing a hemodynamic model to estimate the hidden neuronal states (Li et al., 2012). In our study, the model evidence suggests that the optimal models for all three networks were the whole-connected model. For the EB subjects, there were bidirectional connections between the bilateral IPC and PCC, mPFC and left IPC, as well as the mPFC and right IPC, which was consistent with previous causal studies on the effective connectivity within the DMN (Zhou et al., 2011; Xu et al., 2017). We also discovered directed connections between the PCC and mPFC, which are both functionally and anatomically connected, and play a crucial role in the DMN.

Inconsistent findings on the coupling between regions within the DMN have been reported in many studies. Using the stochastic DCM, our recent study found an influence from the PCC on the mPFC (Xu et al., 2017). Some other studies using GCA showed an mPFC to PCC effective connection. Inconsistent with our findings, bilateral IPC were observed to be capable of modulating mPFC and PCC (Di and Biswal, 2014). Furthermore, reciprocal connections between bilateral IPC were observed in the present study, as suggested in another fMRI study (Li et al., 2012). In our study, bilateral IPC showed a slight functional asymmetry and exerted causal influences on the PCC and mPFC, but not vice versa. Therefore we can assume that bilateral IPCs possess a modulating or driving role, as confirmed by a previous study (Di and Biswal, 2014). However, some inconsistent results were reported. Using GCA, the directed connection from the left IPC to mPFC was observed for EB subjects, whereas there were symmetrical connections from the bilateral IPC to PCC, as well as the connectivity from the mPFC to bilateral IPCs (Jiao et al., 2011). This discrepancy may be caused by the smaller sample sizes in previous studies, which lead to several connections not being significant.

In addition, the approach of the spectral DCM in this study is crucial, as it can elucidate the neuronal connectivity abnormalities underlying the functional correlations of fMRI signals and provide more insight into the organization of the functional networks (Birnbaum and Weinberger, 2013). Our DCM analysis suggested that the mPFC appeared to be a zonal area, as there were separate efferent and afferent connections between the mPFC and other regions, which was of particular interest given its role in personal evaluation, choice behavior and reality monitoring (Rushworth et al., 2004; Metzak et al., 2015). The function of the mPFC has been proposed to manipulate the correlations between learning associations between different actions, context and adaptive responses (Euston et al., 2012). Moreover, one functional connectivity study has further demonstrated that signals in the mPFC may be responsible for the signal integration between mPFC and other brain regions, such as the posterior superior temporal cortex and AI (Hare et al., 2010). Using a multimodal MR-imaging technique, decreased functional connectivity were observed between the parietal and frontal areas (Bauer et al., 2017), while this reduced effective connectivity were also discovered in the DMN in the EB subjects.

### Effective Connectivity Analysis Within Other Functional Brain Networks

The left AI has played a critical role not only in the semantic and language network (Binder and Desai, 2011), but has also been considered as a “supramodal convergence zone” (Binder et al., 2009; Binder and Desai, 2011), which is involved in the association network and information integration from several sensory modalities. Decreased effective connection between right AI and other regions were observed in our study, as shown in Figure 5. The right AI has been confirmed to be a functional hub in coordinating brain networks to produce adaptive behavior, especially the modulation in the DMN and DAN (Wang et al., 2018), as suggested in our study. A weakened functional connection in the early deprivation subject was discovered between the subregions of AI (Nomi et al., 2016; Liu et al., 2017), as the reduced connection from left AI to right AI in our study. Abnormalities within the IPL have been associated with working memory dysfunction as well as impairments in cognitive insight and reality perception (Guo et al., 2014; Lee et al., 2015; Chahine et al., 2017). A recent research based on connectivity-based parcellation of the left inferior parietal lobule suggested that this region was functionally connected to areas more strongly involved in the higher level of social cognitive and language processes, as opposed to more rostro-ventral areas of the IPL that appeared to be associated with the lower functionality level (Bzdok et al., 2016). Our findings suggest that the specific abnormalities of the AI and general alterations within the IPL may be mediated by inhibitory interactions from other regions of the left dorsal attention network. A recent study in EB subjects also suggested that early visual deprivation can selectively reshape the functional architecture of the salience network (Liu et al., 2017). Our study found that most of the effective connections were decreased in the EB subjects, suggesting a corresponding reorganization of the human brain due to the early deprivation.

There are several limitations to our study. First, our analysis does not allow us to conclusively determine the functional significance of these correlations. This study displayed low average positive symptomatology which possibly precluded us from finding additional correlations and, more importantly, our resting-state experiments were not accompanied by the working memory, executive functioning or language-related behavioral paradigms, so that we could obtain more functionally specific correlations. Second, the sample size of both the EB patients and normal control groups was relatively small and moderate. More qualified subjects should be recruited in future studies to increase the reliability of our observations and the effect of the causal interactions in the DMN, SN, and DAN. This study showed preliminary findings and further studies are needed to retest our discoveries in other blind cohorts. Finally, we did not examine the changes of the effective connectivity within all the brain networks, especially the primary sensory networks like the visual network. The purpose of this study is to see if there were disruptions in the high-order cognitive networks that are associated with the cognitive behaviors in the EB subjects. And the Group ICA could not separate the visual cortex into small parcellations. In future studies, we could examine the dynamic changes of EB subjects using other techniques that could parcellate the visual cortex. In this study, we tested the hypothesis that there was a disruption of the effective connectivity in the cognitive networks. The cognitive networks may functionally connected as a whole, thus the future direction will focus on the causal interactions between the cognitive networks besides the directed connections between regions.

## Conclusion

In this study, fully connected models were identified to be the optimal model for the effective connections within the intrinsic functional brain networks in EB subjects. Statistical analyses suggested significant differences in the effective connectivity within the DMN, SN, and DAN between EB and HC groups. More reduced effective connections within three networks by comparing the EB to HC groups indicated that the interactions within the high-order brain networks got greatly suppressed due to the early deprivation. Our analysis additionally revealed the neural connective abnormalities that may underlie alterations of the intrinsic brain network in EB subjects.

## Data Availability

The datasets generated for this study are available on request to the corresponding author.

## Author Contributions

MZ and JT designed the experiments. XL, AW, JiX, and PW performed the experiments. XL, JuX, and ZS analyzed the data. XL, JuX, and ZS wrote the manuscript. BW, MZ, and JT revised the manuscript.

## Conflict of Interest Statement

The authors declare that the research was conducted in the absence of any commercial or financial relationships that could be construed as a potential conflict of interest.

## Footnotes

1 http://www.fil.ion.ucl.ac.uk/spm/

2 http://icatb.sourceforge.net/
